# Neonatal screening for four lysosomal storage diseases with a digital microfluidics platform: Initial results in Brazil

**DOI:** 10.1590/1678-4685-GMB-2017-0227

**Published:** 2018-06-04

**Authors:** Eurico Camargo, Jaqueline Schulte, Jamile Pereira, Heydy Bravo, Claudio Sampaio-Filho, Roberto Giugliani

**Affiliations:** 1 Centro de Triagem Centro de Triagem Porto AlegreRS Brazil Centro de Triagem, Porto Alegre, RS, Brazil; 2 Hospital de Clínicas de Porto Alegre Hospital de Clínicas de Porto Alegre Serviço de Genética Médica Porto AlegreRS Brazil Serviço de Genética Médica, Hospital de Clínicas de Porto Alegre, Porto Alegre, RS, Brazil; 3 Universidade Federal do Rio Grande do Sul Universidade Federal do Rio Grande do Sul Programa de Pós-graduação em Genética e Biologia Molecular Porto AlegreRS Brazil Programa de Pós-graduação em Genética e Biologia Molecular, Universidade Federal do Rio Grande do Sul, Porto Alegre, RS, Brazil; 4 Intercientífica Intercientífica São José dos CamposSP Brazil Intercientífica, São José dos Campos, SP, Brazil; 5 Universidade Federal do Rio Grande do Sul Universidade Federal do Rio Grande do Sul Departamento de Genética Porto AlegreRS Brazil Departamento de Genética, Universidade Federal do Rio Grande do Sul, Porto Alegre, RS, Brazil

**Keywords:** Lysossomal storage diseases, neonatal screening, digital microfluidics, Brazil

## Abstract

We describe the initial results of a neonatal screening program for four lysosomal storage diseases (MPS I, Pompe, Gaucher and Fabry) using the digital microfluidics methodology. The method successfully identified patients previously diagnosed with these diseases and was used to test dried blood spot samples obtained from 10,527 newborns aged 2 to 14 days. The digital microfluidic technology shows potential for a simple, rapid and high-throughput screening for these four diseases in a standard neonatal screening laboratory.

Neonatal screening for lysosomal storage diseases (LSDs) has been gaining considerable interest as a result of the new policies to promote early diagnosis of rare diseases, the development of new screening methods, and the availability of enzyme replacement and other specific therapies for several LSDs. Also, LSDs are not considered too rare anymore, with a combined incidence for the around 50 LSDs estimated to be around 1/7,000 (Meikle *et al.*, 1999; Poorthuis *et al.*, 1999).

LSDs have a wide spectrum of clinical signs and symptoms, with overlapping findings across different diseases and with most disorders presenting large variability (Gieselmann, 2005). This makes the diagnosis particularly difficult, especially as it needs to be confirmed with sophisticated biochemical and genetic tools (Wang *et al.*, 2011). For these reasons there is often a delay in the diagnosis of LSDs (Vieira *et al.*, 2008). This delay may influence the outcome of treatment, already available for several of these conditions (Giugliani *et al.*, 2016).

The development of high-throughput protocols with multiplexing capabilities for use with dried blood spot (DBS) samples has facilitated the establishment of NBS programs for LSDs around the world (Spada *et al.*, 2006; Hwu *et al.*, 2009; Hopkins *et al.*, 2015).

We present here the initial results obtained with a fluorimetric digital microfluidics (DMF) platform used for the neonatal screening of four LSDs [Mucopolysaccharidosis I (MPS I), Gaucher, Fabry, and Pompe diseases].

A DMF platform was used to assay the activities of α-L- iduronidase (IDUA), to screen for MPS I; acid α-glucosidase (GAA), to screen for Pompe; acid β-glucosidase (GBA), to screen for Gaucher; and acid α-galactosidase (GLA), to screen for Fabry in the newborn’s samples.

We used one workstation with four digital microfluidic instruments to run a multiplexed fluorometric enzymatic assay platform as described previously (Sista *et al.*, 2011; Sista *et al.*, 2013). All necessary hardware and reagents for GAA, GBA, GLA, and IDUA were supplied by Baebies Inc (Durham, NC, USA).

DBS samples from patients previously diagnosed with MPS I, Gaucher, Pompe or Fabry diseases were initially tested to check the capability of the method in identifying the respective enzyme deficiencies (Table 1).

**Table 1:** Averages for each enzyme activity in normal subjects, cut-off values and results in confirmed cases previously diagnosed.

MPS-1	Results (μmol/L/h)
Samples (n=9864)	
IDUA average	17.1
calculated IDUA cut-off (30%)	5.1
Positive samples (n=3)	
Patient 1	3.4
Patient 2	4.9
Patient 3	3.7
FABRY	Results (μmol/L/h)
Samples (n=9988)	
GLA average	18.9
calculated GLA cut-off (30%)	5.7
Positive samples (n=2)	
Patient 1	4.2
Patient 2	4.7
Patient 3	3.6
POMPE	Results (μmol/L/h)
Samples (n=9560)	
GAA average	19.8
calculated GAA cut-off (30%)	5.9
Positive samples (n=2)	
Patient 1	3.4
Patient 2	3.1
GAUCHER	Results (μmol/L/h)
Samples (n=9878)	
GBA average	13
calculated GBA cut-off (30%)	3.9
Positive samples (n-2)	
Patient 1	3.3
Patient 2	3.5

DBS samples were then collected from 10,527 newborns aged 2 to 14 days of life, randomly selected for testing among the cards routinely received by the Neonatal Screening Center, based in Porto Alegre, Brazil. Samples from newborns were spotted on the filter paper and shipped at room temperature to the reference laboratory, arriving there within 24 to 72 hours .Tests were performed no more than 48 hours after sample arrival.

The DMF assay protocol to determine the enzyme activity in a multiplex format took less than four hours, allowing three runs per day (eventually, one overnight) and yielded 152 results in each run (total, 456 per day) when using one working station with four fluorimeters in parallel. Overall coefficient of variation (CV) values between cartridges, days, instruments, and operators ranged from 4 to 22%. Linearity correlation coefficients were ≥ 0.98 for all assays.

Cutoff values of 5.1 μmol/L/h for IDUA, 5.9 μmol/L/h for GAA, 3.9 μmol/L/h for GBA, and 5.7 μmol/L/h for GLA were established during a preliminary phase using 1,000 samples from the cards routinely collected and DBS specimens from patients with confirmed LSDs previously diagnosed. These cut-off values were not very different from the ones established by Hopkins *et al.* (2015), using the same method (4.0, 8.0, 4.5, and 5.5 μmol/L/h, respectively).

The method correctly discriminated nine samples of affected patients (3 from MPS I, 2 from Pompe, 2 from Gaucher and 2 from Fabry cases) previously diagnosed, from samples of normal subjects. Further information on cutoffs and results in known patients is displayed in Table 1.

Four samples out of the 10,527 tested showed activity below the cutoff and were further investigated until a final conclusion was established. These cases were studied by another group and eventually classified as pseudodeficiency (low activity of enzyme without generation of storage and with no clinical consequences, usually found in patients who present mutations already known as related to pseudodeficiency) of α-L-iduronidase carriership for MPS I (1 case), pseudodeficiency of α-glucosidase (1 case) and carriership for Gaucher disease (1 case) (Bravo *et al.*, 2017).

The availability of effective therapies for several LSDs (Aronovich *et al.*, 2015) and the evidence that early introduction of therapy may bring a better outcome (Gabrielli *et al.*, 2016) make neonatal screening for these conditions an option to be considered. This is especially true as several high-throughput platforms became available to perform tests in a large number of samples at a low cost per assay (Matern *et al.*, 2015).

In the present report we demonstrated that DMF was efficient in identifying samples from patients previously diagnosed with MPS I, Gaucher, Fabry and Pompe diseases. Although we cannot discard that false negatives could occur, this possibility is considered very small, as affected patients usually have extremely low enzyme activities, and all affected patients tested were identified by the method in the validation process (data not shown). The usefulness of this platform was already reported by Hopkins *et al.* (2015), who detected 1/1,618 newborns affected by one of these four diseases in 43,686 samples evaluated. We identified four samples among the 10,527 DBS from newborns tested with a low enzyme activity (1/2,631). Although subsequent testing informed that these were not true LSDs, all these cases had true low enzyme activity, which was detected by the screening program using the DMF method.

This is the first report of neonatal screening for multiple LSDs conducted in Brazil. It is important to mention that this program was performed in a standard clinical biochemistry laboratory, indicating that, in addition to be efficient and robust, the platform is suitable to less sophisticated laboratories, as are most of the laboratories in Latin America. The successful use of this strategy to simultaneously measure four enzyme activities indicates that this method could be an option for Brazil and other developing countries.
